# Identification of Risk Factors for Breast Cancer for Women in Istanbul

**DOI:** 10.2174/1874434600701010006

**Published:** 2007-08-30

**Authors:** Sevim Çelik, Güler Aksoy

**Affiliations:** 1 Zonguldak Karaelmas University, Zonguldak School of Nursing, Surgical Nursing Department, Turkey; 2 Istanbul University, Florence Nightingale College of Nursing, Surgical Nursing Department, Turkey

**Keywords:** Breast cancer, behavioral risk factors, lifestyle changes.

## Abstract

**Background:**

Breast cancer is the most common cancer type seen in women, accounts for 18% of all cancer types in women and the risk of a woman to get breast cancer during her life is 11%. These notified rates enable breast cancer to be defined as a preventable and if pre-diagnosed, a treatable cancer type, despite it was regarded as a terrifying type of cancer in the past.

**Objective:**

The aim of the study was to determine the lifestyle pattern of women without breast cancer in Istanbul.

**Method:**

The study was carried out as a descriptive and cross-sectional study with 1000 women.

**Results:**

The majority of the women (29.7%) were in the 35-44 year old age group. Out of these 93.1% gave birth before the age of 30, 29.5% breastfed for 7-12 months, 65.8% started menarche between 13-15 years of age (mean of 13.3 years), 15.5% were in menopause and had entered menopause at a mean age of 46.5 years. Their mean body mass index was 24.3kg/m^2^ and 24.5% of them preferred foods containing high fat content. The majority of the women (85.4%) did not participate in sports regularly. One third (30.3%) of the women had underwent Breast Self Examination. There was a positive family history of breast cancer for 12.1% of the women.

**Conclusion:**

Sedentary lifestyles, lower Breast Self Examination and routine mammography rates and family histories of breast cancer were the risk factors that needed to be given priority for further action.

## INTRODUCTION

Breast cancer is the most common cancer type seen in women, accounting to about 18% of all cancer types in women. Women have an 11% risk for getting breast cancer during their lifespan [1,2]. Although these reported rates would have been seen as a frightening type of cancer in the past, but today, it is seen as a preventable and, with early diagnosis, treatable type of cancer [3].

In the year 2004, it has been delineated that 371,000 new cases of breast cancer were diagnosed and it was the cause of death for 129,900 women in Europe [4]. It has been predicted that one in every 8 women will experience breast cancer during their lifetime [5,6]. According to data for the year 2005 in Turkey, the rate of breast cancer seen in women was 23.5%, however this rate increased to 24.1%. Breast cancer was primarily diagnosed in the Marmara region in 1999.

A significant proportion of determined risk factors are nonmodifiable factors, such as gender, age, race, family history, late pregnancy, early menarche and late menopause [1,6]. Researchers also note that modifiable factors include postmenopausal obesity from change in lifestyle, cigarette smoking and use of alcohol, physical inactivity, hormone replacement therapy, breastfeeding and reproductive lifestyle behaviors [1,4,7–10]. There are numerous nationally and internationally accepted studies concerning the effect of modifiable and nonmodifiable risk factors on breast cancer incidence [3,4,7,11].

Turkish and Asian women reflect the model of a traditional family, in which a family aims at least two children who are breastfed by the mother. Nutritional habits of Turkish and Asian women show a preference for carbohydrates and fatty diet. Alcohol consumption is relatively restricted in these women due to cultural origin and religious obligations. But lately it has been observed that certain masses of women have started to adopt the western life style giving rise to considerable alcohol consumption and cigaratte smoking. In addition, the sedentary lifestyle of Turkish women has also contributed greatly to an increased risk of breast cancer [22].

This study was conducted for the purpose of determining lifestyle behaviors, which have important roles in preventing and controlling breast cancer in women in Istanbul.

## LITERATURE REVIEW

The incidence of breast cancer is observed in women under the age of 25, with the risk multiplying every ten years till menopause, being particularly most common at post-menopausal period after the age of 50 [12]. The risk of contracting breast cancer for a 20 years old woman in the following 10 years is 0.05%; 0.44% at the age of 30; 1.46% at the age of 40; 2.73% at the age of 50; 3.82% at the age of 60; and 4.14% at the age of 70 [11].

It has been delineated that high education and socioeconomic level increase the risk of breast cancer by enhancing the neural communications within the brain thereby causing an increase in the level of estrogen. Furthermore, it has been highlighted that women being highly educated and maintaining a socio-economic level do have enough knowledge about the benefits of healthy nutrition, consuming which assists them in keeping good health and they start to menstruate at younger ages. Resultantly, this considerable period of time spent in getting educated, followed by exercising significant posts at work, contributes to a delay in marriage and a probable conception and childbirth at older ages, leading to an increased risk of breast cancer [13].

Early onset of menarche (before age 12) and late menopause (after age 50) are associated with increased risk of breast cancer. According to the results of case-controlled studies, the risk of breast cancer reduces per year by 20% for each year the menarche is delayed [12].

Having no children or the first full-term pregnancy by the age of 30 places a woman at an increased risk of breast cancer. When compared with the women whose age at first live birth is 18 or 19, the risk increases 4-5 times for the women being 30 years old at first live birth with this rate being the same for the women who have not given birth. Accordingly, however, it is controversial. Researchers show that hormone replacement therapy for longer than 5 years may increase the risk by 50%-80% at the post-menopausal period [4,10,14, 15].

Studies indicate a close relationship between the duration of breast feeding and the decrease of the risk of breast cancer [4,16]. For women breast feeding for 12 months, the risk of breast cancer reduces by 4.3% and it also reduces by 7% for every birth [4]. Breast feeding is largely protective, as it is especially done for long time periods. When compared with women who never breast feed, it has been determined that the relative risk of breast cancer is 0.95% for those breast feeding for 7-11 months, 0.86% for those breast feeding for 12-23 months, 1.11% for those breast feeding for 24 months and longer. Veronesi *et al.* [4] analyzed the correlation between the duration of breastfeeding and risk of breast cancer. In that study they showed that there is a 4.3% reduction in the risk for women who breastfeed for 12 months with a 7% reduction at each birth.

Seven prospective cohort type reproductive studies (337.819 women and 4385 invasive breast cancer cases) have notified that there is an increase of relative risk of breast cancer related to diets and other risk factors by 1.02% for pre-menopausal women and by 1.07% for post-menopausal women [4].

There are numerous studies that are pointing to the role of regular physical activity on the possibility of decreasing the risk of breast cancer [4,6,17–19]. Friendreich *et al*. [20] noted that inadequate physical activity increases the risk of breast cancer by 1.43%-1.67%. In the study conducted by Verloop *et al.* [21] on women aged between 20 and 54, it is expressed that physical activity in pre-menopausal women decreases the risk of breast cancer by 30%. Researchers are in agreement with the issue that the density and length of physical activity are also effective in decreasing the risk of breast cancer. They emphasized that the risk of breast cancer may decrease for a quite active woman by 10-70% and the risk may lower 30-40% more for a woman doing regular exercise for 3-4 hours per week [6,8,9,12].

Recent studies show that breast cancer is caused by mutations and changes in the genes BRCA I and BRCA 2, and approximately 5-10% of breast cancer cases are hereditary [9,11]. The International Agency for Research on Cancer predicted that the global rate of breast cancer related to obesity and sedentary lifestyle is 25% and noted that the risk increases for post-menopausal women by 50-250% [6].

Recent studies have highlighted the fact that women are becoming more aware about the causes, risk factors, and early diagnosis and treatment of breast cancer, which has also shown to be a positive inclination even in Turkey where a decline in mortality rates has been observed [11].

## METHODS

### Study Design

This study was conducted as a descriptive and cross-sectional study.

### Sample

This study was conducted in Istanbul with a total of 1000 women who were willing to participate. Data revealed the existence of approximately 3,000,000 women over 18 years of age in Istanbul and breast cancer was seen 24.1% in Turkey during the period 2005 and 2006. It was used following the calculation formula:

$\text{n=}\frac{\text{P}.\text{Q}.{\text{Z}}_{\alpha }^{\text{2}}}{{\text{d}}^{\text{2}}}$

*Inclusion criteria* in this study included women who did not have breast or any other gynecological from of cancer, were able to speak Turkish and were over 18 years of age.

*Exclusion criteria* for the study included women diagnosed with breast and any other gynecological from of cancer, women who were unable to speak Turkish or were health professionals. Eleven survey forms were incompletely filled out by participants therefore they were not included in the study.

### Major Study Variables

*Alcohol Consumption:* Respondents reported their alcohol consumption status as yes or no. Quantity of alcohol consumed per month was grouped into four categories: 1-4 glasses, 5-8 glasses, 9-12 glasses and ≥13 glasses.

*Cigarette Smoking:* Participants reported their current cigarette smoking status as yes or no. Quantity of cigarette smoking per day was grouped into five categories: 1-4 cigarettes, 5-9 cigarettes, 10-14 cigarettes, 15-19 cigarettes and ≥ 20 cigarettes.

*Nutritional Habits:* The questions included the women's consumption of fresh fruits and vegetables and the consumption of fatty food.

*Physical Activity:* The participants reported their sports activity and walking status and duration of these activities per day.

*Routine Mammography and Breast Self Examination:* The questions were directed at determining health related behaviors whether the subjects had ever had a routine mammography and Breast Self Examination. In case they had, they reported the time of Breast Self Examination.

### Other Baseline Study Variables

Researches were instructed to maintain records of the patricipants’s including age, age at menarche, education level, marital status, employment status and type of occupation, pregnancy and menopausal status and the use of hormonal replacement therapy.

### Data Collection

A survey form with questions about the risk factors of breast cancer was filled in by 20 women in a pilot test, followed by essential revisions in the form, as a result of which the final survey form included 50-questions.

Study data were collected in collaboration with a clinic practice by research staff. Researhers were assigned to collect information about healthy women through conducting interviews of women who visited the university hospital in Istanbul. Researchers filled out the survey form for the illiterate women after receiving their permission.

### Data Analysis

Data were loaded in the Microsoft Excel software program and evaluated for number and percentages. Chi square tests were conducted to evaluate the relationships between cigarette smoking, alcohol consumption, exercise and nutritional status with age, educational status, marital status and employment status. The confidence interval of 95% was accepted.

### Ethical Approach

All of the hospital directors of nursing, staff nurses and ethics board were informed about the study and their permission was received. The women were informed about the objective of the study and the contents of the survey form and were told that their identity would be kept confidential. These subjects were then asked to give their informed consent.

## RESULTS

### Sample Characteristics

All of the 1000 women in the study were 18 years or older, with 29.7% being in the age group of 35 and 44 years, 33.4% being university graduates, 66.2% were married and 62.6% were employed (Table **1**).

### Reproductive-Related Health Behaviors

The majority (65.8%) of the women started menarche between 13 and 15 years of age, 62.7% had given birth, 93.1% gave birth to their first child before the age of 30 (mean 22.5±4.4) and they breastfed for 7 and 12 months (29.5%). It was noted that 81.5% of the women continued to menstruate, 66.5% of the women who had stopped menstruating had entered menopause under the age of 50 (mean of 46.5±5.7 years), and 19.0% were taking hormone replacement therapy after menopause (Table **2**).

### Health Behaviors Related to Modifiable Risk Factors

About 87.9% of the women had never used alcohol and 73.6% of the alcohol consumers drank 1-4 glasses of alcoholic beverages per month. One third of the women (32.9%) smoked cigarettes and about half of them used a pack or more of cigarettes per day (Table **3**).

On examining the women's nutritional habits, it was determined that all of 61.9% women (619 individuals) ate three meals a day; 84.3% consumed fresh fruits and vegetables, 75.5% low fat products and 77.4% milk and dairy products with meals (Table **3**).

The mean body weight of the women was 63.42 kg and their mean height was 161.64 cm. These results indicated that their mean body mass index was 24.3 (within normal limits). Although only 14.6% of the women reported that they regularly participated in sports activities, 53.4% stated that they walked regularly. Those regularly participating in sports and walking were spending an average of one to two hours a day for those activities (Table **3**).

It was observed that women under the age of 25, though being educated, had an inclination to consume more alcohol. In addition, alcohol consumption was also shown to be higher for employed and single women. There was a statistically, significantly higher percentage of cigarette smoking among women being highly educated and were employed (X^2^: 19.7; p=0.001). The significant differences were found in the comparison of cigarette smoking status with other variables. The rate of cigarette smoking was higher in the women between 35 and 44 years than in the other age groups.

Menopausal women had a lower exercise level than the premenopausal women and the difference was found to be statistically significant. The number of women getting regular exercise was higher at a statistically significant level in the women who were university graduates (X^2^: 17.2; p=0.004), employed (X^2^: 5.44; p=0.02) and married (X^2^: 23.7; p=0.000).

Consumption of a high fat diet was the highest in employed women and among the age interval of 25-34 years. The higher percentage of the university graduates and married women who ate high fat diets was found to be statistically significant (X^2^: 40.6; p=0.000).

### Health Behaviors About Early Detection

The majority of the women in this study (79.7%) did not undergo Breast Self Examination (BSE). Of those who did undergo BSE, 32.5% underwent the procedure a week after the end of menstruation. The majority of the women (97.8%) had not had a mammography test and 84.4% did not get a regular health check-up (Table **4**).

**Fig. (1):**
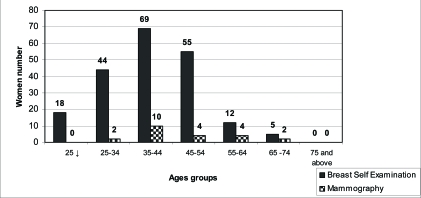
Distribition of women health screening behaviors by ages groups.

The frequency of women getting screening methods decreased at a statistically significant level with increasing age, particularly in the post-menopausal period (Fig. **1**).

About of 12.1% (52 women) had a member of their family presenting with breast cancer.

## DISCUSSION

Many studies have associated the risk of breast cancer to factors such as cigarette smoking and the use of alcohol, nutritional habits, obesity, level of physical activity, menstrual and reproductive characteristics, individual’s level of stress and state of depression, besides sociodemographic characteristics. It has been emphasized that these lifestyle factors may increase the risk of development of breast cancer, or, on the contrary, they may decrease the risk. In this study, based on statements given by women in Istanbul, their lifestyle related behaviors were determined, which can be used to recommend various programs and have an important role in the prevention and control of breast cancer.

### Reproductive-Related Health Behaviors

The incidence of breast cancer is low in women under the age of 25. It however increases every ten years until menopause and increases significantly in the post-menopausal period after the age of 50 [12].

It has been suggested that a high level of education and socioeconomic status may increase the risk of breast cancer. It has been suggested that starting to menstruate at a younger age (before the age of 12), entering menopause before the age of 50 and giving birth to their children at older ages (after the age of 30) increases the risk of breast cancer [9–11,12,23]. Accordingly, although it is controversial, researchers have shown that hormone replacement therapy for longer than 5 years increases the risk by 50-80% in the post-menopausal period [4,10,14,15].

Breastfeeding is an effective factor in the prevention of breast cancer for women who give birth; it decreases the incidence of breast cancer by 25-35% [16]. Studies have also indicated that there is a close relationship between the duration of breastfeeding and a decrease in the risk of breast cancer [4,16].

In this study, the mean age of the women was 36.6 years, with 66.2% being married, 33.4% were university graduates, 62.6% were employed, and the longest place of residence for 53.8% was the city. Menarche manifested between the age of 13 and 15 for 65.8% of the women, 62.7% had given birth and 93.1% of the women who had given birth did so before their 30s (mean age of 22.5); 29.5% breastfed their children for 7-12 months and 26.8% for 13 months and longer, 81.5% of all women were still menstruating, 66.5% of those who were not menstruating any longer had entered menopause before their 50s (mean age of 46.5 years) and 81% of these were not taking hormone replacement therapy after menopause. Our findings indicated that the women living in Istanbul showed behaviors supporting the results of former studies and were not at increased risk from factors related to age, menarche, menopause and age of giving first birth, or the length of breast feeding.

### Health Behaviors Related to Modifiable Risk Factors

There was higher alcohol consumption among young (under the age of 25 and younger, the ages of 25-34), single, working women being highly educated and most of them were consuming 1-4 glasses of alcohol per month. Similarly, the rate of cigarette smoking was found to be higher at a statistically significant level among young and working women being highly educated (a packet or more per day). There is evidence showing that alcohol increases the risk of breast cancer, particularly if used together with cigarettes. There are many studies which report that the age when women start to drink alcohol or smoke (especially under the age of 30) and the quantity of consumption (2 glasses of alcohol per day) have a fundamental role in an increased risk of breast cancer [6,8,11,17,24,25]. However, most of the women in our study were not smoking or using alcohol. Risendal *et al.* [26] reported that women between 30-39 years old, who are single and are highly educated consume more alcohol than normal, but have a lower rate of cigarette use, however, women under 50 years who are single and employed are more active cigarette smokers. Fredman *et al*. [27] and Hunter and O’Dea [28] reported low rates of smoking and alcohol use.

In our study, most of the women had low-fat diets and consumed fresh fruits and vegetables. In particular, the women between 25-34 years who were university graduates and were married stated that they consumed high fat foods. The mean body mass index of the women in our study was within normal limits. An increase of consumption of fast-food was observed in parallel with high level of education, high socio-economic level and a fast-paced work life.

High fatty diets together with alcohol, inadequate consumption of fruits and vegetables, nutritional habits containing low roughage and rich in carbohydrates lead to obesity; consequently, they increase the risk of breast cancer by raising the level of insulin and estrogen in the bloodstream [6,18,26,29,30]. Obesity is regarded as an important factor, which increases the risk of breast cancer in women by 50-250%, however, it is protective in the pre-menopausal period [4,6,31]. In the analysis of 8 cohort studies conducted with 351,825 women, it was determined that the risk of breast cancer had no relationship with the consumption of fruits and vegetables regarding their beneficial effect on decreasing the risk [6]. Malin *et al*. [32] also reported that nutrition mostly with fruit and vegetables could decrease the risk of breast cancer. The literature and the results of former studies suggested that the women in our study at the age of 45 and older presented eating habits, which decreased their risk of breast cancer development, however, the results are rather alarming for the younger women in our study, as they were more likely to face the risk of breast cancer in the following years due to their habits of smoking, using alcohol and eating fatty food.

The results of this study showed that 85.4% of the women living in Istanbul do not participate in sports regularly and 46.6% do not walk regularly. The women with inadequate physical activity were mostly unemployed women with a low level of education and were in their post-menopausal period. Risendal *et al.* [26] also reported that women who are 50 years old and over, have a low level of education and the unemployed have inadequate physical activity in parallel with the findings of our study. Although the results of the study clearly stress the role of physical activity on decreasing the risk of breast cancer, it can be seen that women in our country mostly adopt a sedentary lifestyle. There are numerous studies that point to the role of regular physical activity on the possibility of decreasing the risk of breast cancer [4,6,17–21,33].

Researchers are in agreement that intensity and duration of physical activity are also effective in decreasing the risk of breast cancer. They emphasize that the risk of breast cancer may decrease for a quite active woman by 10-70% and the risk may lower by 30-40% more for a woman who regularly exercises for 3-4 hours per week [6,8,9,12].

### Health Behaviors About Early Detection

One of the risk factors, which women define as the most frightening one, which has an effect on the development of breast cancer is the existence of breast cancer within the family. Recent studies indicate that 5-10% of breast cancer is hereditary and that the cancer is caused by alterations and mutations in the genes, BRCA I and II [8,11,12]. When the existence of any type of cancer or breast cancer in the women's family history was questioned in our study, we determined that the rate was 12.1% for breast cancer. Based on this result, the Istanbul women's positive family histories of breast cancer were characterized as a considerable risk factor. In a different study conducted by Çolak and Korkmaz [34] in Turkey, having a positive family history of breast cancer was reported to be the most serious risk factor. When we examined the women's participation in early methods of detection, it was determined that only 15.6% got a regular check-up, only 20.3% underwent regular BSE and only 2.2% would have a mammography examination. The percentages were even lower in the post-menopausal period. This result emphasize the need to increase mammography and breast self examination among the Turkish women. The literature has emphasized the use of mammography to decrease the risk of death from breast cancer by 30% for women who are over the age of 50 [5,35,36] Madlensky *et al.* [29] noted that women under 40 who have not yet had a mammography screening are a more risky group for cancer. Instead of specifying the top limits, American Cancer Society recommends the age of starting BSE as 20, for breast examination as 20-39 and for mammography as 40 [37]. In the study conducted by Hunter and O’Dea [28] in which they examined how the middle-aged women perceive their risks of health in the future, it was reported that 53% of the women underwent BSE, again the percentage being much higher than the results of our study.

## CONCLUSION AND RECOMMENDATIONS

The women in Istanbul have a tendency to perform healthy behaviors, which aim to decrease or prevent the risk factors presented in the results of the epidemiological study. However, the percentages of those with inadequate physical activity (particularly in non-working woman in their post-menopausal period) and the percentages of women with a family history of breast cancer were high.

The importance of the role of the multidisciplinary approach is undeniable for the prevention and early detection in women at risk for breast cancer [6] Nurses, as members of the multidisciplinary team, need to take part in every process related to breast cancer, including prevention, health education, early detection, treatment and care [11]. Nurses especially should be active about health education and awareness of public. Nurses are expected to guide women, especially in determining women who are at risk and to educate them and encourage them to receive regular screening at available health services [38].

We may conclude that nurses need to emphasize on the sedentary life style, education for BSE and mammography, and give utmost consideration to positive family history in early detection programs and in public awareness programs to prevent breast cancer.

## LIMITATIONS OF STUDY

On examining the demographic data, it was observed that the study sample included women being 40 years old, therefore, it was essential that data should be generalized for such a study. Researhers should also assess housewives and not restrict the age group to 40 years and under, but those over the age of 40 should also be evaluated in the future research.

## Figures and Tables

**Table 1:** Demographic Characteristics of Women

Demographic Characteristics	n	%
*Age* (36.6±12.1) <25 25-34 35-44 45-54 55-64 65-74 ≥75	178 276 297 164 58 19 8	17.8 27.6 29.7 16.4 5.8 1.9 0.8
*Education* Illiterate Literate Primary school Middle school High school University	37 37 253 89 250 334	3.7 3.7 25.3 8.9 25.0 33.4
*Marital status* Married Single Widowed Separated	662 262 64 12	66.2 26.2 6.4 1.2
*Employment status* Employed Unemployed	374 626	37.4 62.6
*Type of occupation* Housewife Labor-intensive work Office type work Freelance Other (student)	374 145 271 63 147	37.4 14.5 27.1 6.3 14.7

**Table 2:** Behaviors About Women’s Reproductive Risk Factors

Reproductive Risk Factors	N	%
*Age of menarche*(13.3±1.3) Cannot remember ≤12 13-15 ≥16	41 238 658 63	4.1 23.8 65.8 6.3
*Pregnancy* Has not given birth Has given birth	373 627	37.3 62.7
*Age at first live birth* (22.5±4.4) (n=627) <30 ≥30	584 43	93.1* 6.9*
*Duration of breastfeeding* (n=627) Did not breastfeed 1-3 months 4-6 months 7-12 months ≥13 months	35 106 133 185 168	5.6* 16.9* 21.2* 29.5* 26.8*
*Menstruation* Menstruating In menopause	815 185	81.5 18.5
*Age of menopause* (46.5±5.7) (n=185) <50 50-55 ≥56	123 57 5	66.5* 30.8* 2.7*
*Hormone replacement therapy* (n=185) Yes No	35 150	19.0* 81.0*

* Percentage calculated according to number (n).

**Table 3:** Behaviors About Women’s Modifiable Risk Factors

Modifiable Risk Factors	N	%
*Consumption of alcohol* Yes No	121 879	12.1 87.9
*Quantity of alcohol consumed (per month),* (n=121) 1-4 glasses 5-8 glasses 9-12 glasses ≥13 glasses	89 20 9 3	73.6* 16.5* 7.4* 2.5*
*Cigarette smoking* Yes No	329 671	32.9 67.1
*Quantity of smoking (per day)* (n=329) 1-4 cigarettes 5-9 cigarettes 10-14 cigarettes 15-19 cigarettes ≥20	52 53 73 11 140	15.8* 16.1* 22.2* 3.3* 42.6*
*Consumption of fresh fruits and vegetables* Yes No	843 157	84.3 15.7
*Consumption of fatty food* Yes No	245 755	24.5 75.5
*Regularly partipicate in sports activity* Yes No	146 854	14.6 85.4
*Duration of sports activity(per day)* (n=146) 1-2 hours 3-4 hours 5-6 hours	131 14 1	89.7* 9.6* 0.7*
*Walking* Yes No	534 466	53.4 46.6
*Duration of walking (per day)* (n=534) 15-29 minutes 30-44 minutes 45-59 minutes ≥60 minutes	40 146 16 332	7.5* 27.3* 3.0* 62.2*

* Percentage calculated according to number (n).

**Table 4:** Behaviors About Early Detection of Breast Cancer of Women

Early Detection Methods	N	%
*Breast Self Examination* Yes No	203 797	20.3 79.7
*Time of Breast Self Examination*(n=203) Whenever it comes to mind A week after menstruation When I have pain With every shower Before and after menstruation Twice a month During menstruation Once a year First day of every month Every fifteenth day of each month	51 66 6 45 8 5 12 8 1 1	25.1* 32.5* 3.0* 22.2* 3.9* 2.5* 5.9* 3.9* 0.5* 0.5*
*Regular check-up* Yes No	156 844	15.6 84.4
*Mammography* Yes No	22 978	2.2 97.8
*Cervical Pap Smear* Yes No	39 961	3.9 96.1

* Percentage calculated according to number (n).

## References

[1] Aydiner A, Dinçer M, Topuz E, Topuz E, Aydiner A, Karadeniz AN (2000). Meme kanseri. Klinik Onkoloji.Istanbul Üniversitesi Onkoloji Enstitüsü Yayinlari.

[2] Stasiolek D, Kwasneiewska M, Drygas W (2002). Breast cancer: Selected risk factors, primary prevention. Przegl Lek.

[3] Yilmaz M, Çetin Z, Karapinar H, Çiftçi S (2005). Cumhuriyet üniversitesinde çalşan kadın personelin meme kanseri risk durumunun belirlenmesi.VIII. Ulusal Meme Hastalıkları Kongresi.

[4] Veronesi U, Boyle P, Goldhirsch A, Orecchia, Viale G (2005). Breast Cancer. Lancet.

[5] Wright T, McGechan A (2003). Breast cancer: new technologies for risk assessment and diagnosis. Mol Diagn.

[6] McTeirnan A (2003). Behavioral risk factors in breast cancer: can risk be modified?. Oncologist.

[7] Emmons KM, Kalkbrenner KJ, Klar N, Light T, Katherine AS, Garber JE (2000). Behavioral risk factors among women presenting for genetic testing. Cancer Epidemiol Biaomarkers Prev.

[8] Korde LA, Calzone KA, Zujewski J (2004). Assessing breast cancer risk: genetic factors are not the whole story. Postgrad Med.

[9] Mertens AJ, Sweeney C, Sharar E, Rosamond WD, Folsom AR (2006). Physical activity and breast cancer incidence in middle-aged women: a prospective cohort study. Breast Cancer Res Treat.

[10] Okobia MN, Bunker CH (2005). Epidemiological risk factors for breast cancer-a review. Niger J Clin Pract.

[11] Sevil Ü, Ünsal Ş (2002). Meme kanserinde risk faktörleri ve erken tanı. Hemşirelik Forumu Dergisi.

[12] Lewis MS, Heitkemper MM, Dirksen SR (2004). Medical Surgical Nursing.

[13] Hilakivi-Clarke L (1997). Estrogen-regulated non-reproductive behaviors and breast cancer risk: animal models and human studies. Breast Cancer Res Treat.

[14] Chen CL, Weiss NS, Newcomb P, Barlow W, White E (2002). Hormone replacement therapy in relation to breast cancer. JAMA.

[15] Pritchart KI (2001). Hormone replacement in women with a history of breast cancer. Oncologist.

[16] Lipworth L, Bailey LR, Trichopoulus D (2000). History of breast-feeding in relation to breast cancer risk: a review of the epidemiologic literature. J Natl Cancer Inst.

[17] Key TJ, Schatzkin A, Willett WC, Allen NE, Spencer EA, Travis RC (2004). Diet, nutrition and the prevention of cancer. Public Health Nutr.

[18] Garrett NA, Brasure M, Schmitz KH, Schultz MM, Huber MR (2004). Physical inactivity direct cost to a health plan. Am J Prev Med.

[19] Rabin CS, Pinto BM, Trunzo JJ, Frierson GM, Bucknam LM (2006). Physical activity among breast cancer survivors: regular exercisers *vs* participants in a physical activity intervention. Psychooncology.

[20] Friendreich CM (2001). Physical activity and cancer prevention: from observational to intervention research. Cancer Epidemiol Biomarkers Prev.

[21] Verloop J, Rookus MA, Kooy K, Leeuwen FE (2000). Physical activity and breast cancer risk in women aged 20-54 years. J Natl Cancer Inst.

[22] Aslan FE, Gürkan A (2007). Kadinlarda meme kanseri risk düzeyi. Meme Sağliği Dergisi.

[23] Butler LM, Potischman NA, Newman B (2000). Menstrual risk factors and early-onset breast cancer. Cancer Causes Control.

[24] Morabia A (2002). Smoking (active and passive) and breast cancer: epidemiologic evidence up to Juna 2001. Environ Mol Mutagen.

[25] Rohan TE, Jain M, Howe GR, Miller AB (2000). Alcohol consumption and risk of breast cancer: a cohort study. Cancer Causes Control.

[26] Risendal B, Dezapien J, Fowler B, Papenfuss M, Giuliano A (1999). Cancer prevention among urban southwestern american indian women: comparison to selected year 2000 national health objectives. Ann Epidemiol.

[27] Fredman L, Sexton M, Cui Y (1999). Cigarette smoking, alcohol consumption, and mammography among women ages 50 and older. Prev Med.

[28] Hunter MS, O’Dea I (1999). Perception of future health risks in mid-aged women: estimates with and without behavioral changes and hormone replacement therapy. Maturitas.

[29] Madlensky L, Vierkant RA, Vachon CM (2005). Preventive health behaviors and familial breast cancer. Cancer Epidemiol Biomarkers Prev.

[30] Stoll BA (2002). Upper abdominal obesity, insulin resistance and breast cancer risk. I J Obes Relat Metab Disord.

[31] Thompson HJ, Zhu Z, Jiang W (2004). Weigh control and breast cancer prevention: are the effects of reduced energy intake equivalent to those of increased energy expenditure?. J Nutr.

[32] Malin AS, Qi D, Shu XO (2003). Intake of fruits, vegetables and selected micronutrients in relation to the risk of breast cancer. Int J Cancer.

[33] Shoff SM, Newcomb PA, Trentham-Dietz A (2000). Early-life physical activity and postmenopausal breast cancer: effect of body size and weight change. Cancer Epidemiol Biomarkers Prev.

[34] Çolak T, Korkmaz B (2005). Meme kanseri risk faktörleri vaka-kontrol çalişması. Ulusal Meme Hastalıkları Kongresi.

[35] Hall HI, Uhler R, Coughlin SS, Miller DS (2002). Breast and cervical cancer screening among Appachian women. Cancer Epidemiol Biomarkers Prev.

[36] Rakowski W, Bren N, Meissner H (2004). Prevalence and correlates of repeat mammography among women aged 55-79 in the Year 2000. Natl Health Interv Survey.

[37] Smith RA, Cokkinides V, Eyre HJ (2003). American Cancer Society Guidelines fort he early detection cancer, 2003. CA Cancer J Clin.

[38] Conto SI, Myers JS (2002). Risk factors and health promotion in families of patient with breast cancer. Clin J Oncol Nurs.

